# Henry Robbins Dew

**Published:** 1911-09

**Authors:** 


### HENRY ROBBINS DEW.

The death of Dr. Dew, one of the oldest practitioners in Bristol, and a Crimean veteran, took place on September 6th, at the age of 75.

He received his professional education at St. George's Hospital, London, became a Member of the Royal College of Surgeons in 1855, and a Licentiate of the Royal College of Physicians of Edinburgh in 1860. He then joined the Army, becoming Staff Assistant Surgeon to Lord Raglan, and serving with distinction through the Crimean War. He was for many years Surgeon to the Deaf and Dumb Institution, the Bristol Lying-in Institution, and the Bristol Eye Hospital. A colleague writes of him as follows:—

"Dr. Dew did good work for many years as Assistant Surgeon to the Bristol Eye Hospital with Dr. Bartley, and in 1882 he became full Surgeon. He was most punctual and regular in his attendance, and was of great service to the Institution at a time when much development in the work done there was taking place. He had a good practical knowledge of diseases of the eye, was a very agreeable and painstaking colleague, and was a skilful operator. In 1887, after twenty years' work at the Hospital, he resigned, but at the desire of the Committee his valuable experience was rendered still available by his election as Consulting Surgeon, which post he retained until the time of his death."

